# Factors that influence the response of the LysR type transcriptional regulators to aromatic compounds

**DOI:** 10.1186/1471-2091-12-49

**Published:** 2011-09-01

**Authors:** Rosa Lönneborg, Peter Brzezinski

**Affiliations:** 1 Department of Biochemistry and Biophysics, Arrhenius Laboratories for Natural Sciences, Stockholm University, SE-106 91 Stockholm, Sweden

**Keywords:** transcriptional regulator, LysR family, inducer specificity, gfp

## Abstract

**Background:**

The transcriptional regulators DntR, NagR and NtdR have a high sequence identity and belong to the large family of LysR type transcriptional regulators (LTTRs). These three regulators are all involved in regulation of genes identified in pathways for degradation of aromatic compounds. They activate the transcription of these genes in the presence of an inducer, but the inducer specificity profiles are different.

**Results:**

The results from this study show that NtdR has the broadest inducer specificity, responding to several nitro-aromatic compounds. Mutational studies of residues that differ between DntR, NagR and NtdR suggest that a number of specific residues are involved in the broader inducer specificity of NtdR when compared to DntR and NagR. The inducer response was also investigated as a function of the experimental conditions and a number of parameters such as the growth media, plasmid arrangement of the LTTR-encoding genes, promoter and *gfp *reporter gene, and the presence of a His_6_-tag were shown to affect the inducer response in *E.coli *DH5α. Furthermore, the response upon addition of both salicylate and 4-nitrobenzoate to the growth media was larger than the sum of responses upon addition of each of the compounds, which suggests the presence of a secondary binding site, as previously reported for other LTTRs.

**Conclusions:**

Optimization of the growth conditions and gene arrangement resulted in improved responses to nitro-aromatic inducers. The data also suggests the presence of a previously unknown secondary binding site in DntR, analogous to that of BenM.

## Background

The family of LysR type transcriptional regulators (LTTRs) is the largest family of bacterial transcriptional factors. They regulate expression of genes involved in a broad range of cellular functions such as amino-acid metabolism, cell division, virulence, nitrogen fixation and degradation of xenobiotics. Common to all LTTRs is a primary structure of approximately 300 amino-acid residues, a C-terminal inducer-binding domain (IBD) and a DNA-binding domain (DBD) in the N-terminal, containing a winged helix-turn-helix motif. A flexible linker region connects the DBD with the IBD. The active form is often a homotetramer [1,2], although recent reports have suggested higher order complexes for some LTTRs such as CrgA and ThnR [3,4].

**Figure 1:**
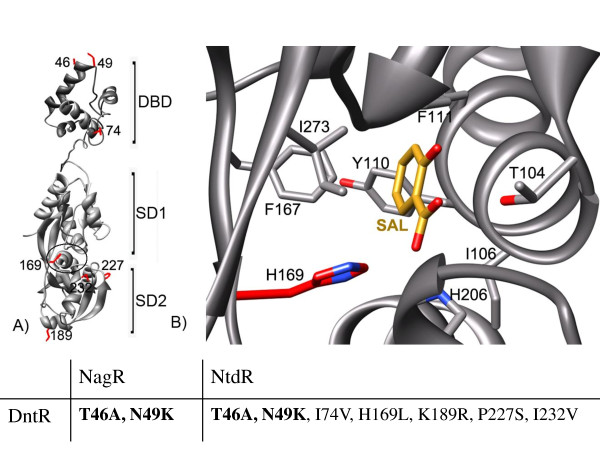
**(a) Overview of one monomer of DntR with the seven residues that are replaced in this study marked in red, with the number of the residue next to it**. Model (see Smirnova *et al*. [8]) of the DNA-binding domain (DBD) and linker connected to the structure of the inducer-binding domain (IBD) are shown as grey ribbons. The previously identified inducer-binding pocket is situated in the region between subdomains 1 (SD1) and 2 (SD2) and its position is encircled (see **b**). One monomer is packed with another monomer in a head-to-tail orientation so that the β-strands seen to the right under the inducer-binding pocket in SD2 forms a parallel β-sheet together with the β-strands seen to the upper left in SD1. (**b**) The inducer-binding pocket shown in detail, with the position of a salicylate (shown in yellow, oxygens marked in red) modelled based on the position of the acetate ion found in the crystal structure. The side-chains of all residues within 4Å of the modelled salicylate molecule are displayed (with oxygens in red and nitrogens in blue, the carbons of His 169 are marked in red). In this model, the hydroxyl group points away from His 169. The His169 residue is the only substituteded residue in this study within 5 Å from the modelled salicylate. Below the pictures, the differences in sequence for NtdR and NagR, compared to DntR, are indicated. In bold are the amino-acid residues in the DBD, while the other five amino-acid residue substitutions are found in the IBD.

A number of full-length structures of members of the LTTR family have been reported; those of ArgP [5], TsaR [6], CbnR [7], and CrgA [4]. Several structures of truncated forms without the DBD are also available, such as those of DntR [8] BenM, CatM [9], CysB [10] and OxyR [11]. The binding site for the inducer is generally situated between the two subdomains in the IBD, although BenM has an additional binding site and OxyR does not appear to bind any ligand. Subdomains 1 and 2 are connected by a flexible hinge region consisting of two antiparallel β-strands that allow the two subdomains to rotate relative to each other (Figure 1). Binding of an inducer is proposed to change the conformation of an LTTR from a repressor state to an activator state, but how this conformational change occurs is still debated [6,9].

For many LTTRs it has been shown that the regulator is always bound to a complex promoter [12-15] that is responsible both for the autorepression of its own gene upstream of the promoter, and repression/activation of metabolic genes downstream of the promoter by activating expression in the presence of an inducer, and in some cases repressing the expression in the absence of the inducer. In other words, the promoter elements for these LTTRs act as two divergent promoters that overlap [16,17] and perform two functions. Typically, the inducers are metabolic intermediates in or substrates for the degradation pathway regulated by the transcription factor.

Several LTTRs, such as NahR [18], DntR [19], NtdR [20,21] and NagR [22], have been reported to regulate the expression of enzymes involved in degradation pathways for aromatic compounds. The three latter transcription factors have almost identical amino-acid sequences (Figure 1) and share about 60% sequence similarity with NahR, which regulates expression of genes needed for degradation of naphthalene via the classical naphthalene pathway [18]. The difference between DntR and NagR are only two amino-acid substitutions in the DBD (99% sequence identity), while the difference between NagR and NtdR are five amino-acid substitutions in the IBD/linker (98% sequence identity) (Figure 1). Salicylate has been reported to be the strongest inducer for all these transcription factors, even though this compound is a metabolite in the degradation pathway only when regulated by NahR or NagR, which both regulate pathways for naphthalene degradation [22]. Both NtdR and DntR were found in strains that are able to degrade nitro-substituted aromatic compounds, where salicylate is not an intermediate. These pathways are likely to have evolved recently from the NagR-regulated naphthalene-degrading pathway, and the sequences of the NtdR or DntR-regulated nitro-aromatic dioxygenase genes are homologous to those of the naphthalene dioxygenase genes, with highest similarity to the naphthalene dioxygenase genes in the NagR-regulated pathway [23]. This recent divergence gives an explanation for the absence of a more sensitive and specific inducer response to nitro-aromatic metabolites for DntR and NtdR.

The inducer specificity has been studied previously for DntR [24], NtdR [25,26] and NagR [27,28] in different strains and with different reporter genes. Somewhat contradictory results were reported. For example, NagR showed a response to 4-methyl salicylate in the former study but not the latter. The most recent study of NtdR [26] investigated the effect on inducer specificity of mutations that differ between NagR and NtdR. The results pointed at certain residues that could be responsible for the broadening of the inducer response observed in NtdR. In the earlier study of NtdR [25], different inducer specificities were observed when NtdR was expressed in different strains, where NtdR showed response to nitro-aromatic compounds only in the strains where it was originally found, and not when expressed in trans in *Escherichia coli *or *Pseudomonas putida*. Also for NagR, different inducer specificities were reported in different studies when other variables in the experimental conditions (such as differences in growth medium and plasmid constructs) than the actual transcription factors were modified. These variations in the results from these studies makes it difficult to conclude whether the different inducer specificities arise as a consequence of actual differences in the molecular mechanisms of these LTTRs, or as a consequence of variations in the experimental conditions.

In the present study, the inducer specificity of DntR, NtdR and NagR was compared under identical conditions, thus generating comparable data. Salicylate and several nitro-aromatic compounds, structurally similar to salicylate or DNT, were tested for inducing capability on these LTTRs using *E.coli *DH5α with a *gfp *reporter gene in fusion with a fragment of the *dntAa *gene, under control of the P_DNT _promoter as a reporter system.

## Results

### The effect of changes in experimental conditions on the LTTR-induced repression and activation of *gfp *transcription

**Figure 2:**
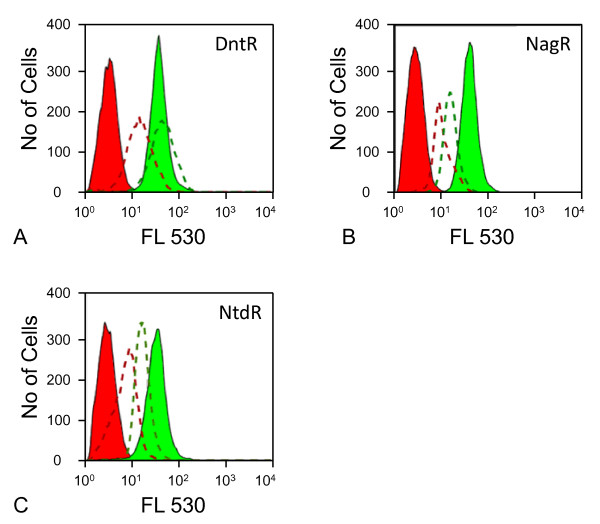
**Histograms showing the cell populations expressing DntR (**a**), NagR (**b**) and NtdR (**c**)**. The fluorescence from the *gfp *reporter gene under the control of the P_DNT _promoter is measured for 10000 cells. The background level of fluorescence where only the solvent DMSO has been added to a cell population harbouring the one-plasmid system (solid red) and DMSO + IPTG added to cells harbouring the two-plasmid system (dashed dark red). The fluorescence level for the cell population with salicylate added for the one-plasmid system (solid green) and for the two-plasmid system (dashed green).

**Figure 3:**
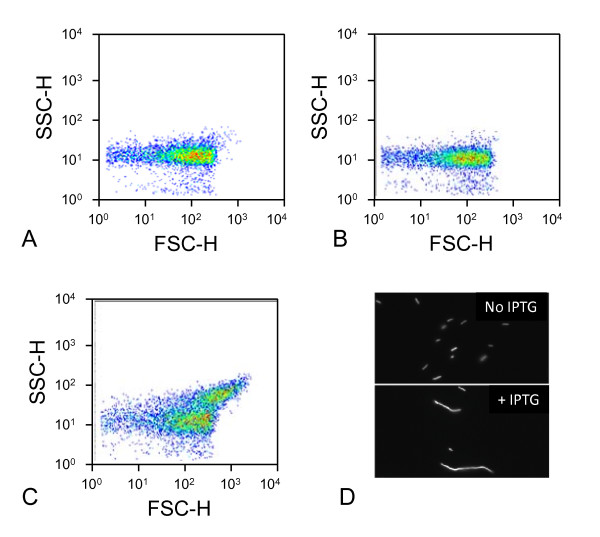
**Example of cell populations with non-filamentous and filamentous cell-growth**. (**a**)-(**c**) shows dotplots for the side scattering and forward scattering for cell populations harbouring the dntR gene. The more filamentous cell growth, the more scattering of light is observed, both in the forward scatter (FSC-H) and side scatter (SSC-H) channels. In (**a**) *E.coli *DH5α harbouring the one-plasmid system and in (**b**), *E.coli *DH5α harbouring the two-plasmid system with addition of only DMSO are shown. Here, the cell population divided normally, and no filamentous cell growth was observed. For the cell population in (**c**), *E.coli *DH5α harbouring the two-plasmid system with addition of DMSO and IPTG, a large subpopulation shows filamentous cell-growth. In (**d**) are shown fluorescence microscope images of the *E.coli *DH5α cells harbouring the two-plasmid system without addition of IPTG (upper picture) and with IPTG (lower picture).

In the absence of an inducer in the *E. coli *DH5α [pQE *LTTR:*P_DNT_:*gfp*] strain (one-plasmid system) where DntR, NagR and NtdR are transcribed from the P_DNT _promoter, the basal level of expression of the *gfp *gene is much lower as compared to when the LTTRs are expressed from the IPTG inducible T5/lac promoter in the *E. coli *DH5α [pQE60 *LTTR*] [pREP P_DNT_:*gfp*] strain (two-plasmid system) (Figure 2). This lower background level of *gfp *expression, contributes to a larger relative effect upon addition of an inducer, such as salicylate, in the one-plasmid system compared to the two-plasmid system. In addition, when adding IPTG to cultures of *E. coli *DH5α harbouring the two-plasmid system to induce expression of the LTTR we observed a considerably slower growth and a high degree of filamentous cell growth. The filamentous cell growth was also observed at IPTG concentrations as low as 10 μM (data not shown). This was not seen with the same strain without IPTG added, nor for the cultures of *E. coli *DH5α harbouring the one-plasmid system, where the levels of LTTR expression are autorepressed (Figure 3). The same type of filamentous cell growth was observed for all the LTTRs, although the degree varied between DntR, NtdR and NagR. To avoid overestimation of the fluorescence we removed the contribution from the filaments by gating the data for the two-plasmid system such that only the sub-population with the same non-filamentous growth as the cell population grown in the absence of IPTG was included. The expression levels of the LTTRs in the two-plasmid system were considerably higher than in the one-plasmid system, with the His6-tag introduced (analyzed by Western blot with an antibody against His_6_-tag as described previously [24], data not shown). The basal fluorescence-levels were also affected by the growth media such that the modified minimal medium gave a lower basal fluorescence level compared to growth in the rich LB medium.

**Figure 4:**
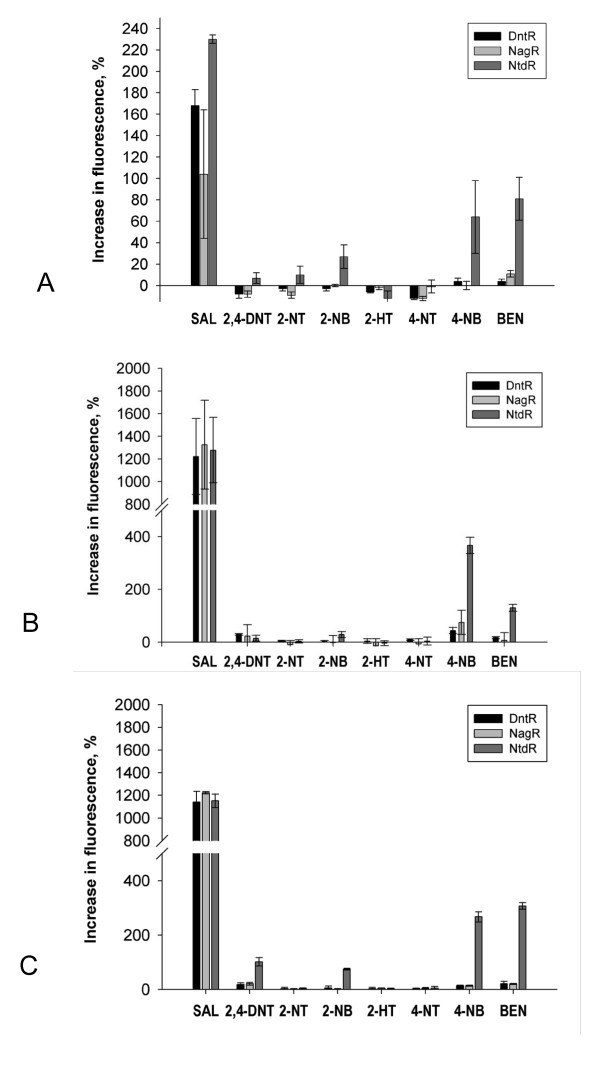
**The inducer response in *E.coli *DH5α for DntR (black bars), NagR (light grey bars) and NtdR (dark grey bars) for three experimental conditions**. (**a**) *E.coli *DH5α harbouring the two-plasmid system for DntR, NagR and NtdR grown in LB. (**b**) *E.coli *DH5α harbouring the one-plasmid system for DntR, NagR and NtdR grown in LB (**c**) *E.coli *DH5α harbouring the one-plasmid system for DntR, NagR and NtdR grown in modified M9 medium. For all conditions the measurements were made 15 hours after addition of the potential inducers. Shown in the diagrams is the increase in fluorescence after addition of the potential inducer, compared to the control culture where only the solvent DMSO (or DMSO+IPTG in A) was added. The mean fluorescence was measured for 10 000 cells in each measurement. All data are based on three independent analyses and shown in error bars are the standard deviations based on these measurements.

For NtdR, DntR and NagR the inducer sensitivity was greatly improved in the one-plasmid system compared to the two-plasmid system (see Figures 2 and 4). For NagR an approximately 9-fold increase in the response to salicylate was observed when the *nagR *gene was expressed from P_DNT _, and for DntR and NtdR a 5-fold increase is seen. This increase in sensitivity when the LTTR is expressed from its own promoter is mainly due to the decreased basal level of expression of the *gfp *gene in the absence of any inducer (see above), but also to some extent to an increased transcriptional activation of the *gfp *gene in the presence of salicylate (in the case of NtdR and NagR) (Figure 2).

The response to 2,4-DNT, benzoate and 2-nitrobenzoate was significantly improved by growth of the one-plasmid system in the modified minimal media compared to LB. However, the sensitivity for salicylate and 4-nitrobenzoate was slightly lowered.

### Comparison of the inducer specificities of NagR, NtdR and DntR

To obtain a measure of the inducer-specificity profile for the three LTTRs, *E.coli *DH5α harbouring the one-plasmid system or the two-plasmid system were grown in the presence or absence of a number of potential inducers in either LB or modified minimal media (Figure 4). The response to salicylate was similar for all three LTTRs in the one-plasmid system and for DntR and NagR there was no significant difference in the inducer-specificity profiles for the other tested compounds. However, NtdR displayed a significantly higher response to 2,4-DNT, 2-nitrobenzoate, 4-nitrobenzoate and benzoate than the two other LTTRs in the one-plasmid system in modified minimal media. This difference in the inducer specificity profile was difficult to distinguish in the less sensitive two-plasmid system for all these compounds, although NtdR displayed a broadened specificity also here.

**Figure 5:**
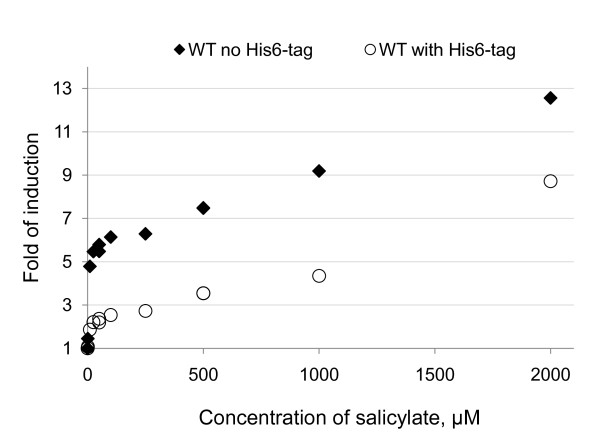
**The response, measured as the fold of induction (mean fluorescence when salicylate is added/mean fluorescence for DMSO control) for salicylate concentrations ranging from 1-2000 μM added to *E. coli *cells harbouring the one-plasmid system grown in LB overnight**. Filled diamonds are the data for wild-type DntR without the His_6_-tag, and open circles are the data for wild-type DntR with the His_6_-tag at its C-terminal.

In the two-plasmid system, a His_6_-tag is introduced at the C-terminal end of the protein. To test whether the His_6_-tag was the reason for the change in sensitivity compared to the one-plasmid system, the His_6_-tagged *dntR *and *ntdR *genes were transferred to the [pQE: P_DNT_: *gfp*] plasmid (used in the one-plasmid system). The His_6_-tag was found to lower the response to salicylate in the one-plasmid system (see Figure 5a), but could not explain the total reduction in response found in the two-plasmid system compared to the one-plasmid system. The His_6_-tag did not cause an increase in basal level of fluorescence in the one-plasmid system to the level found in the two-plasmid system.

**Figure 6:**
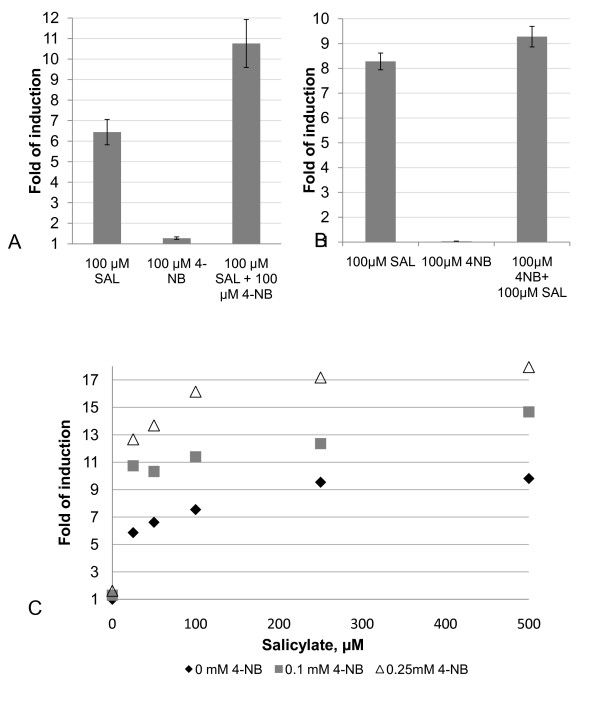
**The effect of addition of 100 μM salicylate (SAL), 100 μM 4-nitrobenzoate (4-NB)or both 100 μM salicylate and 100 μM 4-nitrobenzoate simultaneously to *E. coli *DH5α harbouring WT DntR in the one-plasmid system grown in LB in (a) and grown in M9 in (b)**. In (**c**) the effect is shown for cells grown in LB at 0-500 μM salicylate in the presence of 0 (black filled diamonds), 100 μM (grey squares) and 250 μM (black open triangles) 4-nitrobenzoate respectively. The fold of induction was measured as the mean fluorescence for 10 000 cells with inducer(s) added, divided by the mean fluorescence of 10 000 cells from the control culture with only the solvent added after overnight induction. Data are presented as the mean values based on three independent experiments and shown as error bars are the standard deviations.

The response to the different potential inducers was also followed as a function of time for 22 hours from addition of the potential inducers to cultures of *E.coli *DH5α expressing DntR and NtdR in the one-plasmid system (Additional file 1). When salicylate was added, an increase in fluorescence was observed 1 h after addition, followed by a continuous increase over time. For 2,4-DNT, we observed a small transient response that decreased after a few hours. In the case of 4-nitrobenzoate the increase in fluorescence was slower and could be distinguished after 6 h. As seen in Figure 6a, simultaneous addition of both salicylate and 4-nitrobenzoate for cells grown in the LB medium resulted in an increase in fluorescence (Fold of induction = 10.8 ± 1.2 (SE, 3 measurements)) that was larger than the sum of the effects upon addition of each of these compounds separately (Fold of induction = 7.7 ± 0.6). For the M9 medium the difference was smaller-9.4 ± 0.3 and 8.6 ± 0.7, respectively. Addition of 2,4-DNT, 2-nitrobenzoate or benzoate together with salicylate at the same concentration gave no significant effect compared to salicylate alone (data not shown).

### The effect of mutating residues that differ between NagR, NtdR and DntR

As indicated above, no significant differences were seen between the inducer-specificity profiles for DntR and NagR. These results are not unexpected because only two residues differ between these regulators and the residues are found in the DBD (see Figure 1). NtdR, on the other hand, where five residues differ in the IBD/linker region compared to the two other LTTRs, showed a significantly larger response to 2,4-DNT, 2-nitrobenzoate, 4-nitrobenzoate and benzoate when grown in M9 (In LB, the increase in response was not seen for 2,4-DNT).

All five residues in the IBD of DntR were mutated, one-by-one as well as in various combinations, into the corresponding residues of NtdR. The resulting mutants were analyzed in the two-plasmid system (Additional file 2). A pattern of broadened specificity could be observed in the double mutants H169L/P227S and H169L/K189R. Both these double mutants also had a lower basal level of fluorescence when the mutants were overexpressed compared to WT DntR (estimated as the -IPTG/+IPTG ratio). The P227S mutation alone resulted in higher response to salicylate than wild-type DntR, but no significant response to any of the other potential inducers.

The H169L mutation alone resulted in a very low sensitivity; only a very small response (on the limit of detection) was observed for salicylate and benzoate (and 2-nitrobenzoate). The triple mutant H169L/K189R/P227S displayed a similar induction pattern as for the double mutants H169L/K189R and H169L/P227S, but with lower response levels. The broadening of the inducer specificity is clearly seen in the pentamutant (with all the mutations that differ in the IBD of DntR and NtdR), which shows higher response levels.

When the N49K mutation (in the DBD) was added to the pentamutant, the resulting hexamutant displayed a response to salicylate comparable to that obtained in the one-plasmid system. In addition, a clear response to 2-nitrobenzoate, 4-nitrobenzoate and benzoate and a weaker response to 2,4-DNT could be seen. Also this mutant displayed a lower basal level of fluorescence when the mutant was overexpressed compared to wild-type DntR. The T46A mutation, on the other hand, gave a slightly lowered response to all inducers and in combination with N49K (resulting in NtdR) the repression was reduced and the detected response to the inducers was further lowered.

## Discussion

In the present study the P_DNT _promoter was used in the comparison of the induction profiles of DntR, NtdR and NagR. This promoter was found in the two DntR-expressing strains *Burkholderia sp*. DNT and *Burkholderia *cepacia R34 [29,30] and it is nearly identical to the P_NTD _promoter found in the NtdR-expressing strains *Comamonas *sp. strain JS765 and *Acidovorax *sp. strain JS42 [25], as well as the P_NAG _promoter found in *Ralstonia *sp. strain U2 (for alignment of the promoter regions, see Lessner et al [25]). From the consensus NahR binding region [25] to the -10 region (counted from the *dntA *transcription start) the promoters are identical. Therefore, the binding of DntR, NtdR and NagR to P_DNT_, and to P_NTD _or P_NAG_, is expected to be very similar. Although the four nucleotide changes observed outside the core promoter region might affect the binding affinity, it is assumed not to influence the inducer specificity.

### *E. coli *as a model system for monitoring the inducer responses

In the study by Lessner *et al*. [25] it was reported that the ability of NtdR to respond to nitroaromatic compounds seen in *Comamonas *and *Acidovorax *could not be restored in *E. coli*. In the present study, very little response was seen for some nitroaromatic compounds in *E. coli *DH5α when using similar conditions as in the above-mentioned study (NtdR was expressed in trans, growth in LB medium). However, by optimizing a number of experimental conditions (LTTR expressed from its own promoter, choice of growth medium) the responses to 2,4-DNT, 2-nitrobenzoate, 4-nitrobenzoate, and benzoate were significantly increased for NtdR (see Figure 4). There was no significant response to 2-nitrotoluene or 4-nitrotoluene, as reported for NtdR in the original 2-nitrotoluene-degrading *Acidovorax *strain. This effect could be due to differences in the metabolism or uptake/transport of these compounds between the different bacterial species. The 2-nitrotoluene-degrading *Acidovorax *strain might be capable of accumulating the mono-nitrotoluenes intracellularly, since 2-nitrotoluene is a growth substrate in this strain.

When the fluorescence response was followed over time for the LTTRs expressed in the one-plasmid system in LB, salicylate gave a steady increase over time, while the small response to 2,4-DNT displayed a maximum after a few hours followed by a decrease. In contrast, the response to 4-nitrobenzoate only occurred several hours after addition (Additional file 1). The transient response to 2,4-DNT may be due to reduction of the nitro-groups of 2,4-DNT to the corresponding amine or hydroxylamine, as reported previously for TNT in *E. coli *AB1157 [31]. This is also supported by our observation of an orange colour of the growth media formed in both LB and minimal media a few hours after addition of 2,4-DNT to the bacterial cultures, suggesting the formation of Meisenheimer complexes also observed in the AB1157 strain. The delayed response to 4-nitrobenzoate, on the other hand, may be explained in terms of response to a degradation product of this compound or the presence of an additional binding site for 4-NB that alters the equilibrium of the relative concentrations of the different states of DntR, thereby changing the kinetics of transcriptional repression/activation.

### The additive effect upon addition of both salicylate and 4-nitrobenzoaate

As seen in Figure 6 the response upon simultaneous addition of both salicylate and 4-nitrobenzoate was larger than the sum of responses to each of these compounds. The existence of a second, low affinity site might provide an explanation to this observation. Also, the higher background fluorescence observed in LB compared to the minimal media may be due to an inducing compound(s) that is present exclusively in the LB medium and gives an additive effect together with 4-nitrobenzoate. The additive effect with 4-nitrobenzoate could also be due to a metabolic factor. However, two binding sites have previously been found for another LTTR, BenM, where a synergistic effect was observed upon addition of cis, cis-muconate and benzoate [32]. In the case of BenM, the distinct binding sites have been identified in a truncated form of BenM [9], where the binding site for cis, cis-muconate is situated between the two subdomains in the IBD, analogous to the salicylate-binding site of DntR. The benzoate moiety found in crystals with BenM and cis, cis-muconate was buried in a hydrophobic region of subdomain 2, moving the two subdomains more closely around cis, cis-muconate. Thus, binding of benzoate could provide stabilization of the closed state (less stable with only cis, cis-muconate bound), and thereby increase transcriptional activation. A similar mechanism could be responsible for the effect observed for salicylate and 4-nitrobenzoate with DntR. Recently, new crystal structures of the truncated forms of DntR, co-crystallized with salicylate, were compared with the apo-structure of DntR [33]. Two salicylate moieties bound/IBD monomer were found in a conformation that is suggested to be responsible for full transcriptional activation. In other words, there are two binding sites for aromatic inducers, but it remains to investigate whether or not also 4-nitrobenzoate binds to the other site.

### Effect of mutations that differ between the LTTRs

No significant differences were seen between the inducer-specificity profiles for DntR and NagR, which is not unexpected given that the two residues that differ between these two transcription factors are found in the DBD. In the case of NagR and NtdR, in which the DBDs are identical, while five residues differ in the IBD/linker region, NtdR displayed a significant increase in response to 2-nitrobenzoate, 4-nitrobenzoate and benzoate also in the less sensitive two-plasmid system. Thus, even if the two-plasmid system is less sensitive, the broadening of specificity can be observed when following the response to these compounds where a clear response is seen for NtdR, even though no conclusions can be drawn regarding the changes in response to individual compounds.

When considering mutations that are responsible for the inducer-specificity broadening, our data support the previously obtained results with NtdR mutants, suggesting the key importance of residues 169 and 227 [26] (although different combinations of mutations were constructed in our study compared to that by Ju *et al*.). We choose to focus on combinations of mutations with the H169L mutation, because this residue lines the inducer-binding pocket previously identified in DntR [24], where a F111L/H169V double mutant was shown to respond better to 2,4-DNT than the wild-type DntR. In the study of Ju *et al*. [26], no NagR mutants with H169L in combination with other mutations were analyzed; instead the study was focused on combinations with residues 227 and 232. Thus, our study provides complementary information of the combined effect of H169L with the other mutations.

In the study of Ju *et al*. [26], the single mutations H169L, P227S and I232V in NagR were shown to give the greatest change in inducer specificity, although the H169L mutant had no activity in the assay used. The "opposite" L169H mutation in NtdR, however, resulted in an improved response to nitro-aromatic compounds and an improved repression compared to wild-type NtdR. The H169L mutant of DntR showed very little response to the potential inducers. However, the double mutants H169L/P227S and H169L/K189R displayed a clear broadening of the inducer response that is not seen for the single mutants P227S or K189R alone (Additional file 1). These double mutants were not studied for NagR, instead the double mutant P227S/I232V was shown to recognize a large number of nitroaromatics to which the wild-type NagR was insensitive [26]. The triple mutant I232V/K189R/P227S responded to some additional nitroaromatic compounds compared to the double mutant, indicating that all these residues are involved in modulating the response [26]. Our results with the H169L/P227S and H169L/K189R double mutants suggest that several combinations of the mutations occurring in NtdR (compared to NagR/DntR) can result in a broadening of the inducer specificity. This gives additional possibilities for mutational trajectories towards a response to nitro-aromatic compounds in addition to those suggested by Ju *et al*. [26]. To perform an accurate reconstruction of the evolutionary history of nitroaromatic detection, a complete analysis of all 32 mutants between NagR and NtdR would be necessary, to exclude as many of the 120 possible trajectories as possible. Such a study has been made for β-lactamase, where 102 out of 120 theoretically possible trajectories were found to be inaccessible to Darwinian selection, giving 18 possible paths of protein evolution [34]

### Experimental factors that influence the LTTR-mediated transcriptional regulation of the reporter gene *gfp*

In the present study, we observed that by varying the experimental conditions, such as the composition of the growth media, the background expression levels of the *gfp *reporter changed (and thereby the sensitivity changed). However, the factor that influenced the sensitivity the most was whether the LTTR was expressed from its own promoter in the one-plasmid system or from the T5/lac promoter in the two-plasmid system. The introduction of a His_6_-tag in the two-plasmid system could partly explain the lower response, but could not explain the total reduction of sensitivity.

Previous studies of LTTRs have mainly focused on the regulation of the genes downstream of the promoter, and in many reporter systems, the original gene arrangement has been broken and expressed from a separate plasmid [18,25,28]. Also, the composition of the growth media varies in different studies, where rich growth media have been used in some studies of inducer responses for LTTRs [25,28]. Until now, there have been no comparative studies of how these experimental factors influence the LTTR-mediated response to various inducers.

## Conclusions

In this study, maintaining the original LTTR gene upstream from the dual promoter improved the sensitivity for detecting an inducer response markedly. This is most likely due to the low steady-state level of LTTR expression maintained by the negative autoregulation by its own promoter. This eliminates variations in activity due to expression levels, protein stability and DNA affinity, that is more prominent in the two plasmid system. By improving the growth conditions and gene arrangement, improved responses to nitro-aromatic inducers were obtained for NtdR in *E. coli*, which was not observed previously [25]. Using a plasmid-based system expressed in *E. coli *DH5α rather than a natural isolate as a reporter strain offers several advantages, especially in the field of developing biosensors, where the ease of cloning is important due to the need of improving sensitivity and specificity through mutagenesis [35]. In this study, optimization of growth conditions and plasmid arrangement was shown to increase the sensitivity of such a reporter system. The data also suggest the possibility of a previously unknown secondary binding site in DntR, analogous to that of BenM. Future studies will be focused on the identification of this putative additional binding site.

## Methods

### Chemicals and reagents

Salicylic acid, 2-nitrotoluene, 2-nitrobenzoic acid, 2,4-dinitrotoluene and 2-hydroxytoluene (o-cresol) were all purchased from Sigma-Aldrich. Benzoic acid, 4-nitrobenzoic acid and 4-nitrotoluene were purchased from VWR International. All chemicals were of the highest grade available. For the flow cytometric analysis, 500 mM stock solutions of each compound were prepared in dimethylsulfoxide (DMSO). Restriction enzymes were purchased from Fermentas and T4 DNA ligase was purchased from New England Biolabs.

### Cloning and construction of plasmids

Construction of the pQE60*dntR *plasmid by transferring the *dntR *gene from pLCN60.9 [36] has been described previously [8]. The sequence of *ntdR *and n*agR *was constructed by introducing mutations to the wt *dntR *gene situated in the pQE-60 plasmid according to the manufacturer's instructions with the QuikChange Site-Directed Mutagenesis Kit (Stratagene).

The pREP P_DNT_-*gfp *plasmid was obtained by PCR amplification of the P_DNT_-*gfp *fragment from the vector pLCN60.9 using the forward primer TAT TAT AAA **TTC GAA **CCT CAC CCT and the reverse primer CAT CCG CCA AAC AGC C**AA GCT T**, introducing Hind III sites (bold) at both ends of the P_DNT_-*gfp *fragment. The fragment and the plasmid pREP4 (QIAGEN Nordic) were restricted with Hind III followed by ligation. The pQE60*dntR *plasmid and the pREP4 plasmid together ensure IPTG-inducible expression of the *dntR *gene in what is referred to as the two-plasmid system (Additional file 3a).

The pQE*dntR*-P_DNT_-*gfp *that is used in the one-plasmid system (Additional file 3b) was obtained by digesting the pLCN60.9 plasmid with XhoI and HindIII, producing the *dntR*-P_DNT_-*gfp *fragment which was ligated to pQE-60, digested with the same restriction enzymes. This also resulted in removal of the T5/*lac*-promoter from the plasmid. The *ntdR and nagR *genes were transferred by digesting the pQE*dntR*-P_DNT_-*gfp *and the pQE60*ntdR/nagR *plasmids with EagI and BamHI followed by ligation. The His_6_-tag was introduced into the pQE*dntR*-P_DNT_-*gfp *plasmid by digesting this plasmid and the pQE60*dntR *plasmid with EagI and HindIII so that a *dntRHis_6 _*fragment was transferred into the resulting pQE*dntRHis_6_*-P_DNT_-*gfp *plasmid. The sequences of the LTTR genes and the promoter region were in all cases confirmed by sequencing (MWG biotech, Germany)

### Expression, strains and growth conditions

*E. coli *DH5α cells with or without the plasmid pREP P_DNT_:*gfp *were made electrocompetent according to the following protocol: A 1 L culture of cells in LB media with addition of 25 μg/ml kanamycin were grown to an OD_600 _of 0.5 and then kept on ice for 30 minutes. The cells were washed twice in ice-cold 10% glycerol and kept on ice for 20 minutes between each washing step. They were resuspended in 10% glycerol, and aliquots were frozen in liquid nitrogen and stored at -80°C until they were used.

Prior to each flow cytometric analysis, *E. coli *DH5α[pREP P_DNT_:*gfp*] were transformed with one of the pQE-60 plasmid variants, and *E. coli *DH5α were transformed with one of the pQE *LTTR*-P_DNT_-*gfp *variants. *E.coli *DH5α [pQE60*LTTR*] [pREP P_DNT _:*gfp*] (two-plasmid system) were grown in LB media supplied with 100 μg/ml ampicillin and 25 μg/ml kanamycin at 30°C with shaking. Cells from overnight cultures were used to inoculate new 1 ml cultures with a starting OD_600 _of 0.05. These cultures were allowed to grow for 4 h (OD_600_~0.2-0.3) at 30°C with shaking, and were then diluted with 1 ml LB with 100 μg/ml ampicillin, 25 μg/ml kanamycin and 2 mM IPTG. In the control experiments without IPTG this dilution medium did not contain IPTG. After one additional hour, the various potential inducers (or the solvent DMSO in the control experiments without the inducer) were added to a final concentration of 500 μM. Either 4 h or 15 h later the cells were diluted 1: 500 in PBS prior to FACS analysis.

For comparison with the *E.coli *DH5α [pQE60*LTTR*][pREP P_DNT _*gfp*] strains (two-plasmid system), the *E.coli *DH5α [pQE *LTTR*:P_DNT_:*gfp*] strains (one-plasmid system) were grown in LB or modified M9 (Difco M9 minimal salts (Becton Dickinson) with addition of 1 mM MgSO_4_, 0.1 mMCaCl_2_, 0.2% glucose and 1%LB) media supplied with 100 μg/ml ampicillin at 30°C with shaking. The cells were grown under the same conditions as the *E.coli *DH5α [pQE60*dntR/ntdR/nagR*][pREP:P_DNT_- gfp] strains, but no kanamycin or IPTG was supplied.

### Flow cytometric analysis

The flow cytometric analyses were performed on a FACS Calibur instrument (BD Biosciences, San Jose, CA, USA). Fluorescence was detected via a 530 ± 15 nm (green) band-pass filter. The same instrument settings were used throughout the measurements, and a gate was set to remove contribution from filamentous cells. For each cell sample, data from 10 000 events within the gate was collected. Flow cytometric data were analysed using the FlowJo software, and the mean fluorescence intensity for each cell population was measured. For the analyses with the two-plasmid system, cells grown with addition of only DMSO and cells with addition of 1 mM IPTG and DMSO served as controls. All fluorescence intensities were normalized to the fluorescence intensity of the control (with IPTG and DMSO), analyzed at the same occasion. For the analyses with the one-plasmid system, cells grown with addition of DMSO served as control.

## Abbreviations

IBD: inducer binding domain; DBD: DNA-binding domain; LTTR: LysR type transcriptional regulator; SAL: salicylate; 2,4-DNT: 2,4-dinitrotoluene; 2-NT: 2-nitrotoluene; 2-NB: 2-nitrobenzoate; 2-HT: 2-hydroxytoluene; 4-NT: 4-nitrotoluene; 4-NB: 4-nitrobenzoate; BEN: benzoate.

## Authors' contributions

RL and PB designed the experimental strategy for this study and RL performed the experiments. RL and PB analyzed and interpreted the data, and wrote the manuscript. Both authors have read and approved the final manuscript.

## Supplementary Material

Additional file 1 **The response to some aromatic inducers followed over time**. The response, measured as the GFP fluorescence for whole cells, upon addition of DMSO, salicylate, 2,4-DNT or 4-nitrobenzoate, were followed over time (addition of 500 μM at t = 0). The cells were grown in LB and in A), the strain *E.coli *DH5α [pQE*NtdR*: P_DNT_: *gfp*] was analyzed and in B) the strain *E.coli *DH5α [pQE *DntR*: P_DNT_: *gfp*] was analyzed. Click here for file

Additional file 2 **Table showing the response to some aromatic inducers for a number of DntR mutants**. The inducer response for mutants of DntR with amino-acid substitutions that are found in NtdR. The analysis was performed in the two plasmid system grown in LB, 20 h after induction. Responses are expressed as the % increase in fluorescence after addition of the listed inducer, compared to the fluorescence for the same mutant with addition of only the solvent DMSO. The -IPTG/+IPTG column lists the change of fluorescence when no overexpression of the LTTR variant occurs (no IPTG added) compared to when the LTTR variant is expressed (1 mM IPTG added). Click here for file

Additional file 3 **Schematic overview of the plasmids used in this study**. **A**) "The two plasmid system": plasmid pQE60*LTTR *to the left and pREP P_DNT _*gfp *to the right. **B**) "The one plasmid system": the plasmid pQE *LTTR*-P_DNT_-*gfp*. The complete sequence for the pQE60 plasmid that is used for both the pQE60wtDntR and the pQE*dntR*:P_DNT_:*gfp *constructs and the sequence for pREP are available from Qiagen. Click here for file
