# Supplementary figures and images for: Factors that influence the response of the LysR type transcriptional regulators to aromatic compounds
Source: BMC Biochem. 2011 Sep 1;12:49. doi: 10.1186/1471-2091-12-49 (PMC3180648; doi:10.1186/1471-2091-12-49)

## Slide 1
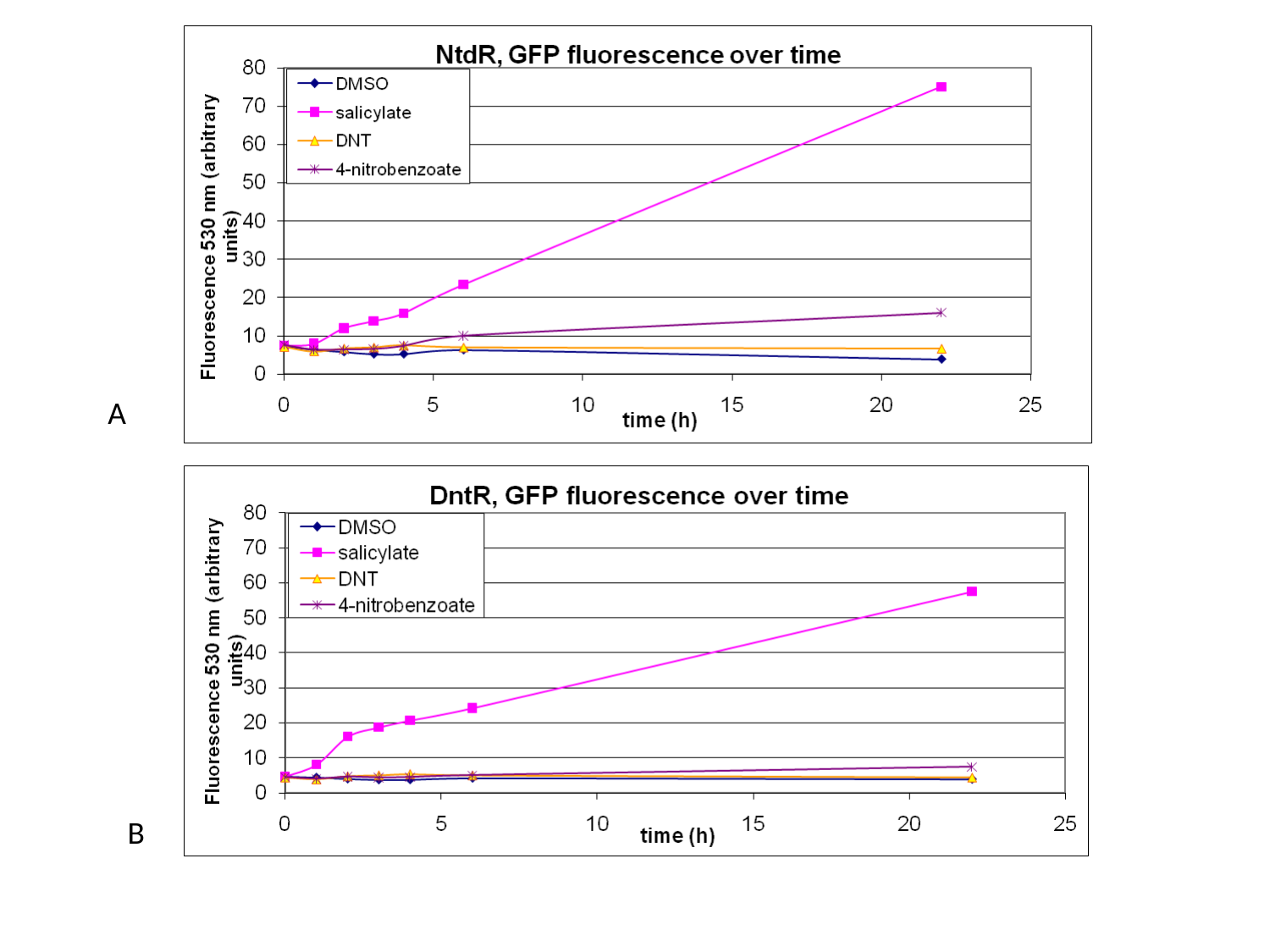

A
B

Supplement: Additional file 1 — The response to some aromatic inducers followed over time. The response, measured as the GFP fluorescence for whole cells, upon addition of DMSO, salicylate, 2,4-DNT or 4-nitrobenzoate, were followed over time (addition of 500 μM at t = 0). The cells were grown in LB and in A), the strain E.coli DH5α [pQENtdR: PDNT: gfp] was analyzed and in B) the strain E.coli DH5α [pQE DntR: PDNT: gfp] was analyzed. [file 1471-2091-12-49-S1.PPT]

## Slide 1
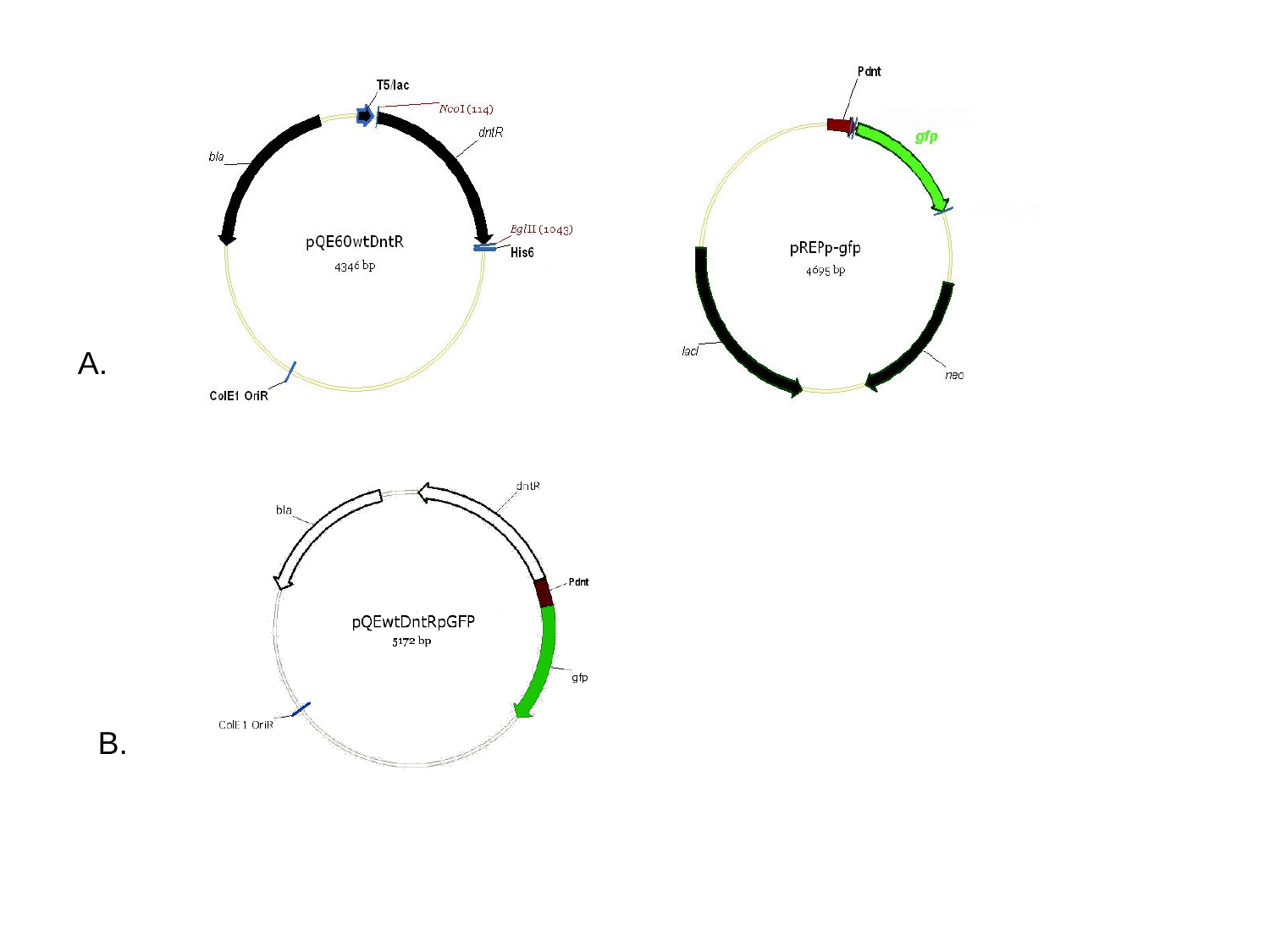

A.
B.

Supplement: Additional file 3 — Schematic overview of the plasmids used in this study. A) "The two plasmid system": plasmid pQE60LTTR to the left and pREP PDNT gfp to the right. B) "The one plasmid system": the plasmid pQE LTTR-PDNT-gfp. The complete sequence for the pQE60 plasmid that is used for both the pQE60wtDntR and the pQEdntR:PDNT:gfp constructs and the sequence for pREP are available from Qiagen. [file 1471-2091-12-49-S3.PPT]
