# Supplementary material for: Factors that influence the response of the LysR type transcriptional regulators to aromatic compounds
Source: BMC Biochem. 2011 Sep 1;12:49. doi: 10.1186/1471-2091-12-49 (PMC3180648; doi:10.1186/1471-2091-12-49)
Supplement: Additional file 2 — Table showing the response to some aromatic inducers for a number of DntR mutants. The inducer response for mutants of DntR with amino-acid substitutions that are found in NtdR. The analysis was performed in the two plasmid system grown in LB, 20 h after induction. Responses are expressed as the % increase in fluorescence after addition of the listed inducer, compared to the fluorescence for the same mutant with addition of only the solvent DMSO. The -IPTG/+IPTG column lists the change of fluorescence when no overexpression of the LTTR variant occurs (no IPTG added) compared to when the LTTR variant is expressed (1 mM IPTG added). [file 1471-2091-12-49-S2.PPT]

## Slide 1
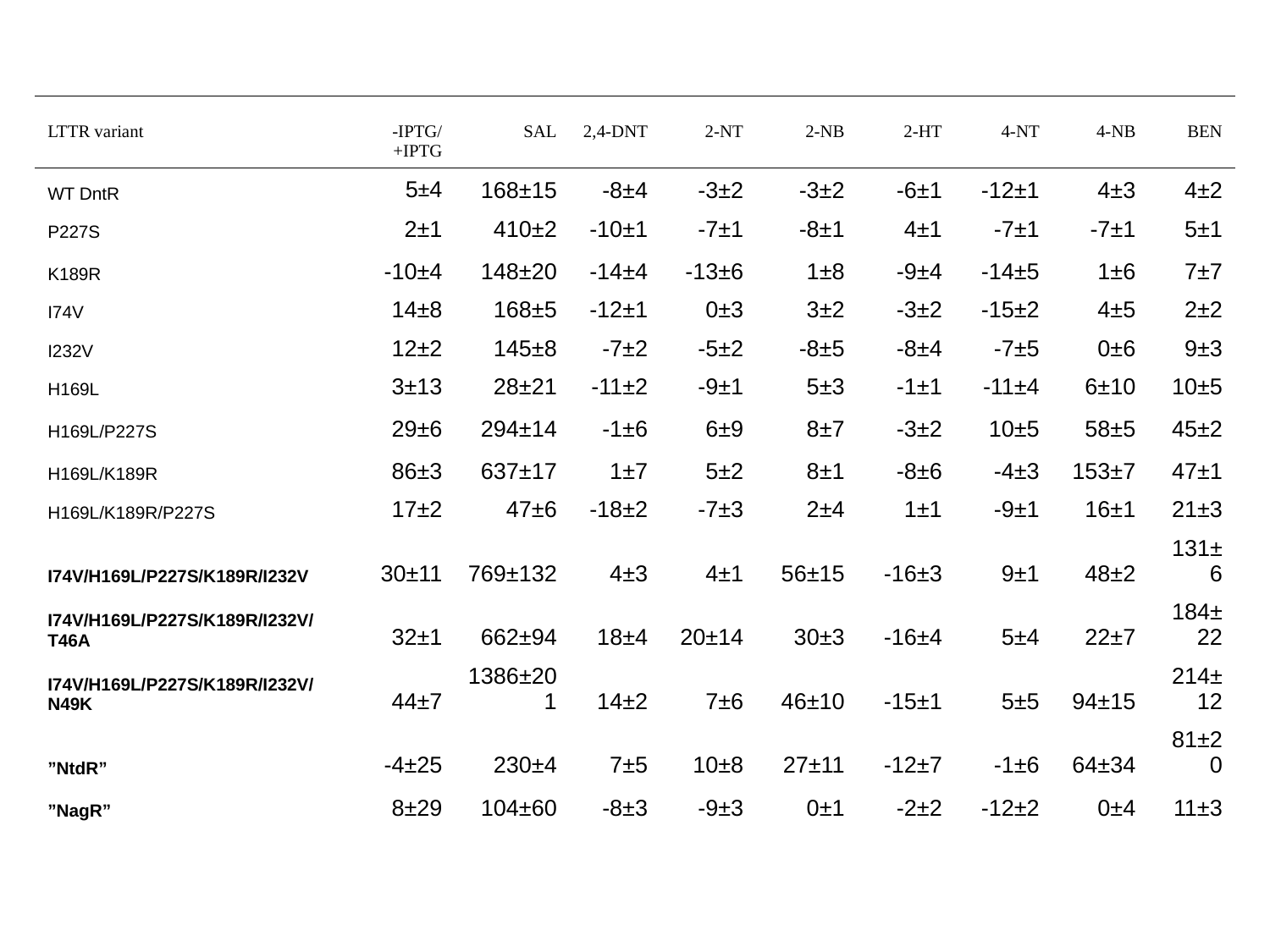

| LTTR variant | -IPTG/+IPTG | SAL | 2,4-DNT | 2-NT | 2-NB | 2-HT | 4-NT | 4-NB | BEN |
| --- | --- | --- | --- | --- | --- | --- | --- | --- | --- |
| WT DntR | 5±4 | 168±15 | -8±4 | -3±2 | -3±2 | -6±1 | -12±1 | 4±3 | 4±2 |
| P227S | 2±1 | 410±2 | -10±1 | -7±1 | -8±1 | 4±1 | -7±1 | -7±1 | 5±1 |
| K189R | -10±4 | 148±20 | -14±4 | -13±6 | 1±8 | -9±4 | -14±5 | 1±6 | 7±7 |
| I74V | 14±8 | 168±5 | -12±1 | 0±3 | 3±2 | -3±2 | -15±2 | 4±5 | 2±2 |
| I232V | 12±2 | 145±8 | -7±2 | -5±2 | -8±5 | -8±4 | -7±5 | 0±6 | 9±3 |
| H169L | 3±13 | 28±21 | -11±2 | -9±1 | 5±3 | -1±1 | -11±4 | 6±10 | 10±5 |
| H169L/P227S | 29±6 | 294±14 | -1±6 | 6±9 | 8±7 | -3±2 | 10±5 | 58±5 | 45±2 |
| H169L/K189R | 86±3 | 637±17 | 1±7 | 5±2 | 8±1 | -8±6 | -4±3 | 153±7 | 47±1 |
| H169L/K189R/P227S | 17±2 | 47±6 | -18±2 | -7±3 | 2±4 | 1±1 | -9±1 | 16±1 | 21±3 |
| I74V/H169L/P227S/K189R/I232V | 30±11 | 769±132 | 4±3 | 4±1 | 56±15 | -16±3 | 9±1 | 48±2 | 131±6 |
| I74V/H169L/P227S/K189R/I232V/T46A | 32±1 | 662±94 | 18±4 | 20±14 | 30±3 | -16±4 | 5±4 | 22±7 | 184±22 |
| I74V/H169L/P227S/K189R/I232V/N49K | 44±7 | 1386±201 | 14±2 | 7±6 | 46±10 | -15±1 | 5±5 | 94±15 | 214±12 |
| ”NtdR” | -4±25 | 230±4 | 7±5 | 10±8 | 27±11 | -12±7 | -1±6 | 64±34 | 81±20 |
| ”NagR” | 8±29 | 104±60 | -8±3 | -9±3 | 0±1 | -2±2 | -12±2 | 0±4 | 11±3 |
